# P-1408. Beyond the Transplant: Understanding CMV Disease in Minority Kidney Recipients

**DOI:** 10.1093/ofid/ofae631.1583

**Published:** 2025-01-29

**Authors:** M Gabriela Cabanilla, Ashlee Dauenhauer, Briana St John, Ngoc-Yen Pham, Deirdre Hill, Joshua Larson, Pooja P Singh

**Affiliations:** University of New Mexico Health Sciences Center, Albuquerque, New Mexico; University of New Mexico Health Sciences Center, Albuquerque, New Mexico; University of New Mexico Health Sciences Center, Albuquerque, New Mexico; University of New Mexico Health Sciences Center, Albuquerque, New Mexico; University of New Mexico Health Sciences Center, Albuquerque, New Mexico; University of New Mexico Health Sciences Center, Albuquerque, New Mexico; University of New Mexico Health Sciences Center, Albuquerque, New Mexico

## Abstract

**Background:**

Disparities in kidney transplant evaluation for minorities raise concerns about post-transplant care access, including cytomegalovirus (CMV) infections and their impact. While CMV significantly affects morbidity and mortality rates, the precise ramifications of these disparities remain unclear. We aimed to evaluate CMV infection patterns in minority kidney transplant recipients.

**Methods:**

We evaluated CMV infection in minority kidney transplant recipients using retrospective analysis and qualitative surveys. Patients aged ≥ 18 years with post-transplant CMV infection were included. Data were collected from University of New Mexico Health Sciences Center records and provider surveys. The primary outcome was CMV disease incidence among minority populations, with secondary outcomes exploring clinical endpoints and barriers to CMV management. Quantitative and qualitative data were analyzed using descriptive statistics, chi-square and Fisher’s exact tests, and thematic analysis.

**Results:**

Among 58 patients (84.5% minorities), CMV disease cumulative incidence was 12.3% in minorities vs. 0% in non-Hispanic whites (p = 0.58), despite similar prophylaxis. No significant CMV disease risk factors were found. Graft failure occurred in 8.6% of patients, mostly minorities, with one graft loss after CMV diagnosis. Mortality appeared higher in minorities (24.4% vs 11.1%, p = 0.67), especially within 90 days of CMV diagnosis, and notably elevated in diabetics (40% vs 9.1%, p = 0.01).

Eight respondents (75% transplant specialists) highlighted CMV management challenges from diagnosis to resource access. Providers acknowledged limitations in current strategies and expressed optimism for newer therapies, such as letermovir. Systematic barriers, including policy gaps and suboptimal multidisciplinary collaboration, were identified as hindrances to comprehensive care delivery.

**Conclusion:**

Our study underscores the CMV disease burden among minority kidney transplant recipients, highlighting disparities in incidence and clinical outcomes compared to non-Hispanic whites. Urgent interventions and policy reforms are required to address systemic barriers and improve post-transplant care equity, ultimately enhancing the health outcomes of minority transplant patients.

**Disclosures:**

**M. Gabriela Cabanilla, PharmD, PhC**, Merck & Co: Grant/Research Support

